# STRAIN: an R package for multi-locus sequence typing from whole genome sequencing data

**DOI:** 10.1186/s12859-019-2887-1

**Published:** 2019-11-22

**Authors:** Mattia Dalsass, Margherita Bodini, Christophe Lambert, Marie-Cécile Mortier, Marco Romanelli, Duccio Medini, Alessandro Muzzi, Alessandro Brozzi

**Affiliations:** 1 GSK, Siena, Italy; 2 Present address: Department of Experimental Oncology, European Institute of Oncology, Milan, Italy; 3 GSK, Rixensart, Belgium; 4 Università degli studi di Siena, Siena, Italy

**Keywords:** Whole genome sequencing (WGS), Multi locus sequence type (MLST), Sequence type (ST), R package

## Abstract

**Background:**

Multi-locus sequence typing (MLST) is a standard typing technique used to associate a sequence type (ST) to a bacterial isolate. When the output of whole genome sequencing (WGS) of a sample is available the ST can be assigned directly processing the read-set.

Current approaches employ reads mapping (SRST2) against the MLST loci, k-mer distribution (stringMLST), selective assembly (GRAbB) or whole genome assembly (BIGSdb) followed by BLASTn sequence query.

Here we present STRAIN (ST Reduced Assembly IdentificatioN), an R package that implements a hybrid strategy between assembly and mapping of the reads to assign the ST to an isolate starting from its read-sets.

**Results:**

Analysis of 540 publicly accessible Illumina read sets showed STRAIN to be more accurate at correct allele assignment and new alleles identification compared to SRTS2, stringMLST and GRAbB. STRAIN assigned correctly 3666 out of 3780 alleles (capability to identify correct alleles 97%) and, when presented with samples containing new alleles, identified them in 3730 out of 3780 STs (capability to identify new alleles 98.7%) of the cases. On the same dataset the other tested tools achieved lower capability to identify correct alleles (from 28.5 to 96.9%) and lower capability to identify new alleles (from 1.1 to 97.1%).

**Conclusions:**

STRAIN is a new accurate method to assign the alleles and ST to an isolate by processing the raw reads output of WGS. STRAIN is also able to retrieve new allele sequences if present. Capability to identify correct and new STs/alleles, evaluated on a benchmark dataset, are higher than other existing methods. STRAIN is designed for single allele typing as well as MLST. Its implementation in R makes allele and ST assignment simple, direct and prompt to be integrated in wider pipeline of downstream bioinformatics analyses.

**Electronic supplementary material:**

The online version of this article (10.1186/s12859-019-2887-1) contains supplementary material, which is available to authorized users.

## Background

Multi-locus sequence type (MLST) is a standard technique used to associate a sequence type (ST) to a bacterial isolate. An ST is a numeric code assigned to a unique combination of allele variants for a small set of housekeeping genes (commonly seven or eight, depending on the species). Sequence typing methods are essential epidemiological tools in infection prevention and control for discriminating different bacterial isolates of the same species. A review of MLST methods has been published by Maiden et al. in 2013 [1].

The broad availability of high throughput sequencing technologies (HTS) makes bacterial whole genome sequencing (WGS) feasible in research and clinical laboratories.

In the near future, it is likely that WGS will replace currently locus-wise typing methodologies due to its limited costs and high throughput readout. However, the analysis of short reads, the common output of sequencing technologies, still represents an open issue.

So far, two families of methods have been described to assign ST from short reads. The standard approach is the whole genome assembly of the short reads that produce “contigs” spanning the entire genome sequence. These contigs, are used to query a reference database of deposited known alleles of the housekeeping loci to identify perfect matches and determine the allele sequence harbored by the isolate which must be typed. Such allele assignment procedure is implemented by the BIGSdb application [2], which is the reference web application tool for the assignment of alleles and ST identifiers. One major advantage of this procedure is that it is able to return new allele sequences if present. A non-negligible disadvantage is represented by the fact that the genome assembly is time-consuming and many parameters must be tuned to achieve satisfactory quality levels.

In 2014, Inouye et al. proposed SRST2 [3], a tool for the detection of genes, alleles and multi-locus sequence types (MLST) based on the mapping of the entire set of short reads against the reference allele database. This approach uses a statistical method to determine the likelihood of alleles without assembling short reads. SRST2 has been proved to outperform the assembly-based approaches because it is computationally faster and more sensitive. A disadvantage is that in case of indels, the new allele consensus sequence is not included in the output FASTA files and ad hoc assembly of the reads is needed.

More recently an assembly- and alignment-free, platform-independent program called *stringMLST* has been released [4]. The program implements a simple hash table data structure to find exact matches between short sequence strings (k-mers) and an MLST allele library. An advantage of stringMLST is that it is very rapid. However, it does not report the sequences of new alleles.

In 2016 a new program named GRAbB (Genomic Region Assembly by Baiting) [5] was developed and released by researchers of the Fungal Biodiversity Centre (Utrecht, the Netherlands). GRAbB is dedicated to assembling specific genomic regions from NGS data.

Here we present STRAIN: a hybrid strategy between assembling-only and mapping-only methods for ST assignment. STRAIN is implemented as an R package, this analysis environment being used in the bioinformatics field. STRAIN is parallelized, reducing the computational time on modern computers and provides higher level of accuracy. We conceived STRAIN i) to overcome the whole-genome assembly step, focusing only on the loci of interest, ii) to improve capability to identify correct STs, iii) to improve the capability to reliably identify new alleles and finally iv) to infer the sequences of new alleles in case they are present in the sample.

### Implementation

**Fig. 1:**
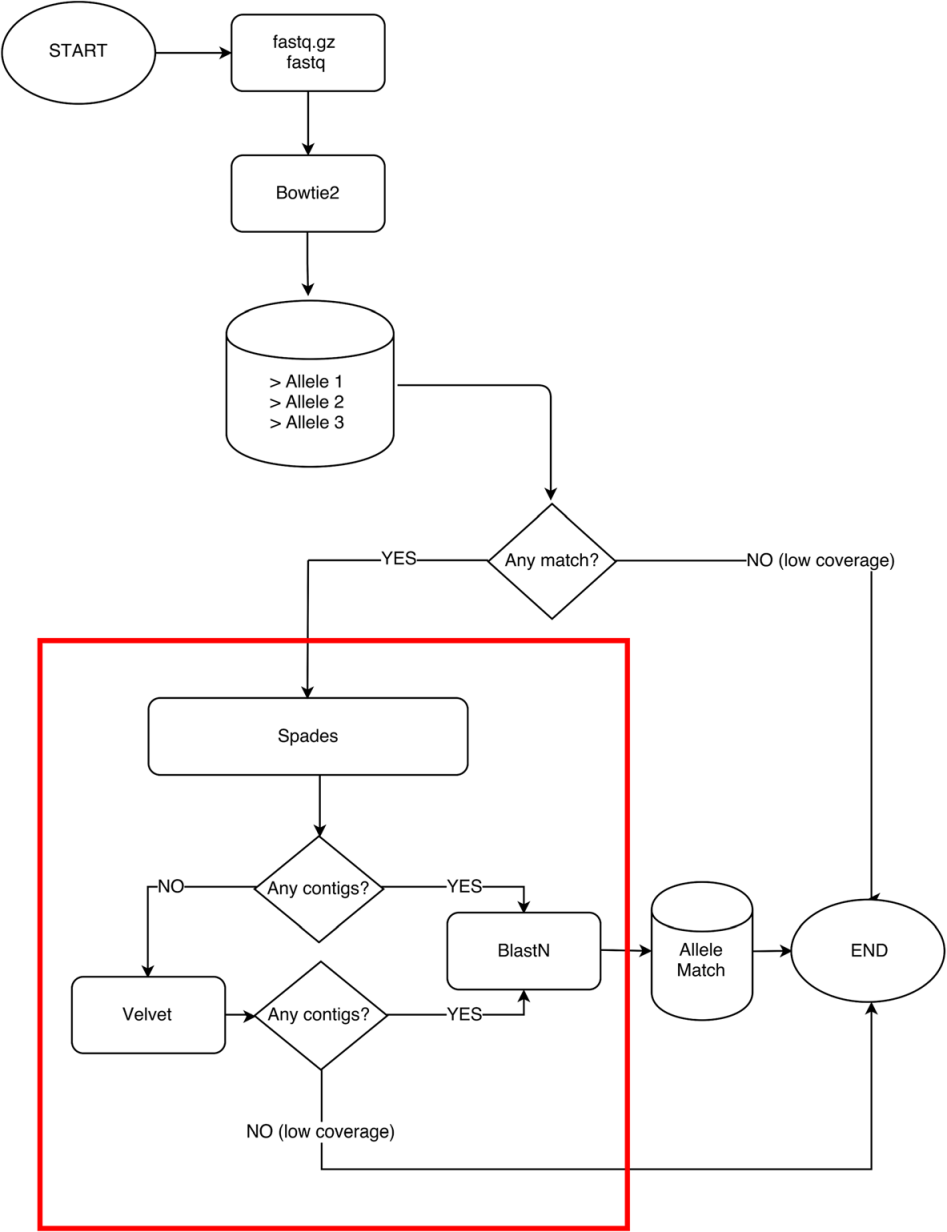
Summary of STRAIN pipeline. The program starts with input sequence file(s) in FASTQ format (compressed or not) and a dataset of reference allele sequences in FASTA format. Using bowtie2, reads are mapped against the reference alleles dataset. Four separate sets of mapped reads are formed according to an increasing match criterion (all mapped reads, reads perfectly matching at least 30, 60 and 90% of their lengths). As highlighted in the red box, each set of reads is separately assembled by SPAdes or Velvet to produce the locus contig(s). Each contig is finally aligned by BLASTn against the reference alleles dataset to identify the perfect match. The program stops i) when there are no reads to be assembled, ii) when no contigs are produced by the assemblers, iii) a new allele variant sequence is identified or iv) a perfect match is found

An overview of STRAIN implementation is depicted in Fig. 1. The idea of our methodology is to reconstruct the sequences of each locus of interest by assembling the subset of reads that map, either partially or totally, to the reference databases of its known alleles.

In the first step bowtie2 [6] is used to map the entire set of reads against the allele sequences of the locus. Bowtie2 is run against the allele variants of each of the seven loci of interest. The output of bowtie2 is saved in seven different files, one for each locus. We use bowtie2 to filter the reads that match the seven loci of interest from the reads that match the rest of the genome. We run bowtie2 with low stringency parameters to allow the program to return a good number of matching reads. Such matching reads can align for different percentages of their length to the reference alleles. We apply three filters on the minimal percentage of read alignment in order to test increasing informative matchings.

The second step (red square in Fig. 1) consists in the assembly performed by SPAdes [7] of the subset of reads. At the end, if the resulting contig(s) perfectly matches for the entire length only one known allele with no mismatches and no indels, we assign the isolate to that allele variant. If the subset of reads assembles in contig(s) shorter, longer or different (with mismatches or indels) to any deposited allele we do not assign the isolate to any known allele and the program returns a message “New?”, envisioning the presence of a novel variant. A directory called “new alleles” is created and contains the sequences of the putative new alleles. Instead, if the number of reads is insufficient for the assemblers to create a contig (low coverage) the program returns a message “low coverage”.

#### Algorithm description

STRAIN is designed to be run on a standard computer and requires only minimal information: the path to a directory containing the gzipped FASTQ files (fastq.gz) generated by the isolate WGS experiment and a path to a folder containing the reference FASTA files of the known allele sequences for the loci of the MLST profile. For species annotated in PubMLST the FASTA files together with the profile definition can be downloaded using the function getMLST() designed to dump the sequences directly from the public repository PubMLST (http://pubmlst.org/).

FASTQ files are merged into a single file disregarding read pairing, i.e. both forward and reverse reads are merged together in a single file (if sequencing consisted of one million paired-end reads 150 bps long, the merged file will be of two million reads 150 bps long). Each locus is processed separately in parallel. All reads are mapped against known allele sequences using bowtie2 (version 2.2.6 and above) program with *–very-fast-local* to handle soft clipped reads. Direct assembly of these reads can introduce a variety of allele chimeras catching non-specific reads especially at the border of the locus. To overcome this problem all mapped reads are also filtered to generate 3 additional sets:

1. reads perfectly matching at least 30% of their lengths;
2. reads perfectly matching at least 60% of their lengths;
3. reads perfectly matching at least 90% of their lengths.

These four sets (all, 30, 60 and 90%) are assembled separately by SPAdes (version 3.6 and above) [7] with the options --k-mer 11, 15, 21, 33, 35 and --only-assembler. To overcome the cases in which SPAdes may fail to reconstruct a contig because of high GC, low or uneven coverage (SPAdes documentation: http://cab.spbu.ru/software/spades/) we set another assembler, Velvet [8], as backup step.

Then each resulting contig is aligned to the reference database of known alleles using BLASTn (version 2.3.0 or higher) looking for any perfect match. If none of the contigs perfectly match a specific allele a self-explaining message “New?” is reported and its sequence stored in a directory called “new alleles”.

## Results

To assess the performance of STRAIN we used the same dataset published in Inouye et al., 2014 [3]. The 543 sets of reads were downloaded in FASTQ format from the EBI SRA public database (ftp://ftp.sra.ebi.ac.uk/) in December 2017. Out of these 543 isolates, 2 samples of *E. faecium* ST 187 were not available because they were not deposited in SRA, and the sample *S. sonnei* ST152 ERR025725 was duplicated (personal communication). For the sample NC_007384, reads were simulated like in the original dataset using simNGS with a coverage of 150x. Therefore, the reference benchmark dataset was made up of 540 samples (Additional file 1). The ST being composed by 7 loci, the total number of allele calls is 540*7 = 3780.

### Definitions of the performance metrics used to compare STRAIN to other tools

We defined three performance metrics:

1. Percentage of no-output.
2. Capability to identify the correct alleles.
3. capability to identify new alleles.

Extended definitions of 1), 2) and 3) are the following.

Percentage of no-output: it is the percentage of times a program reports a blank output. The lower the percentage of no-output the better the performance of the program.

A blank output or NA (Not Available) might happen when the reads in the sample are few or uninformative.

Capability to identify correct alleles: is the fraction of times a program infers an allele variant equal to the correct one. This measure goes from 0% (inferred allele variants are always wrong because different from the correct ones) to 100% (allele variants are always right because equal to the correct ones).

Our benchmark dataset of 540 samples was analyzed trough PCR or capillary sequencing as reported in Inouye et al., 2014 [3]. Allele variants and the STs assigned through these experimental assays are considered as the correct ones.

Capability to identify new alleles: is the fraction of times a program returns a new allele sequence or an indication that a new allele is present when testing against a reference dataset of alleles from which we removed the correct allele sequences.

If a bacterial isolate contains an allele sequence not yet deposited in the reference dataset, a good program should be in fact able to ascertain that the sample manifests a new allele. To measure this capability, one could proceed by genetically engineering bacterial isolates substituting an allele sequence with a completely new one and then performing the sequencing and analyses of the reads. An alternative procedure is, on the other hand, to keep the reads of the sample as they are and to remove from the set of reference allele sequences the alleles assigned by PCR. A program that tested on this modified reference set infers always the presence of a new allele has a capability to identify new alleles of 100%. On the contrary a program that returns always another allele of the reference set will have a capability to identify new alleles of 0%. We therefore created for each sample a reference allele dataset lacking the alleles assigned by PCR. The predictions of the programs are in this case right when they report the new allele sequence or an indication that a new allele is present; wrong when they report another allele of the reference.

The ST is given by the combination of the seven allele predictions. As just described for alleles, we define capability to identify correct ST the fraction of times in which the ST predicted by a program is equal to the correct ST (assessed by PCR or capillary sequencing); capability to identify new STs the fraction of times in which the predicted ST is new when testing with a reference set of alleles missing the correct ones.

### Evaluation of the output of stringMLST and SRST2

As reported in the documentation, when the output of SRST2 (version 0.2) is in the form of “allele *”, it indicates that one or more of the best scoring alleles have at least one mismatch (SNP or indel, according to majority rules consensus of the aligned reads vs the allele sequence). This often means that a new allele is present.

For stringMLST, depending on its parameter called fuzzy (default 300), the output “allele *” indicates potentially new alleles, like SRST2.

Accordingly, we set for both programs two different levels of stringencies based on the interpretation of the output “allele *”:

- High stringency**: “**allele *” is considered as an indication that a new allele is present.
- Low stringency: “*” is ignored and the “allele” is considered the program prediction.

For example, suppose that sample A has by PCR “allele_1” for the locus *adk* which in total has 100 different alleles. “allele_1” is considered the correct one. Table 1 exemplifies the different cases for capability to identify the correct alleles and capability to identify new alleles at stringency levels high and low.

**Table 1:** Interpretation of the output of stringMLST and SRST2 with “*”

	Capability to identify correct alleles		Capability to identify new alleles	
SRST2 / stringMLST output	High stringency	Low stringency	High stringency	Low stringency
allele_1*	Missed the correct allele	Hit the correct allele	*(this case doesn’t exist because allele_1 is not present)*	*(this case doesn’t exist because allele_1 is not present)*
allele_2* to allele_100*	Missed the correct allele	Missed the correct allele	Hit a new allele is present	Missed a new allele is present

Synoptic table describing differences in the interpretation of output containing “*” with high and low stringency criteria for both capability to identify correct and new alleles. To measure the capability to identify new alleles, in this example “allele_1” has been removed from the set of reference alleles

### STRAIN outperforms the other tested programs in the capability to identify correct alleles

Table 2 shows a summary of the percentage of no-output and capability to identify correct alleles for STRAIN and the other tools. For detailed running setup of the tools see Additional file 2.

**Table 2:** Capability to identify correct alleles

Software	Percentage of no-output	Capability to identify correct alleles
STRAIN	0.6% (22/3780)	**97.0% (3666/3780)**
SRST2 high stringency	1.8% (69/3780)	94.8% (3583/3780)
SRST2 low stringency	1.8% (69/3780)	96.9% (3662/3780)
stringMLST high stringency	0.3% (9/3780)	28.5% (1076/3780)
stringMLST low stringency	0.3% (9/3780)	96.9% (3664/3780)
GRAbB	2.7% (101/3780)	92.6% (3499/3780)

Summary of percentage of no-output and capability to identify correct alleles for STRAIN, SRST2, stringMLST and GRAbB. In bold the maximum value

STRAIN show higher capability to identify correct alleles in respect to all other methods. The improvement against SRST2 goes from 0.1 to 2.2% considering the two filters low or high respectively and it is even wider against stringMLST, from 0.1 to 68.5% (low and high stringency). Against GRAbB the improvement is 4.4%. Consequently, STRAIN delivers improvement also at ST level (Additional file 2: Table S1).

### STRAIN outperforms the other tested programs in the capability to identify new alleles

As shown in Table 3, STRAIN outperforms the other tools also in terms of their capability to identify new alleles with a minimum difference of 1.6% compared to GRAbB (98.7% versus 97.1%) to a maximum difference of 98.7% compared to stringMLST low stringency (98.7% versus 0%). The advantage in respect to GRAbB is also that STRAIN has a lower percentage of no-output (0.6% versus 2.7%).

**Table 3:** Capability to identify new alleles

Software	Percentage of no-output	Capability to identify new alleles
STRAIN	0.6% (22/3780)	**98.7% (3730/3780)**
SRST2 high stringency	1.8% (69/3780)	85.9% (3247/3780)
SRST2 low stringency	1.8% (69/3780)	1.1% (41/3780)
stringMLST high stringency	0.3% (12/3780)	80.5% (3057/3780)
stringMLST low stringency	0.3% (12/3780)	0% (0/3780)
GRAbB	2.7% (101/3780)	97.1% (3669/3780)

Summary of percentage of no-output and capability to identify new alleles for STRAIN, SRST2, stringMLST and GRAbB. In bold the maximum value

Moreover, STRAIN improves the capability to identify new STs (Additional file 2: Table S2).

**Fig. 2:**
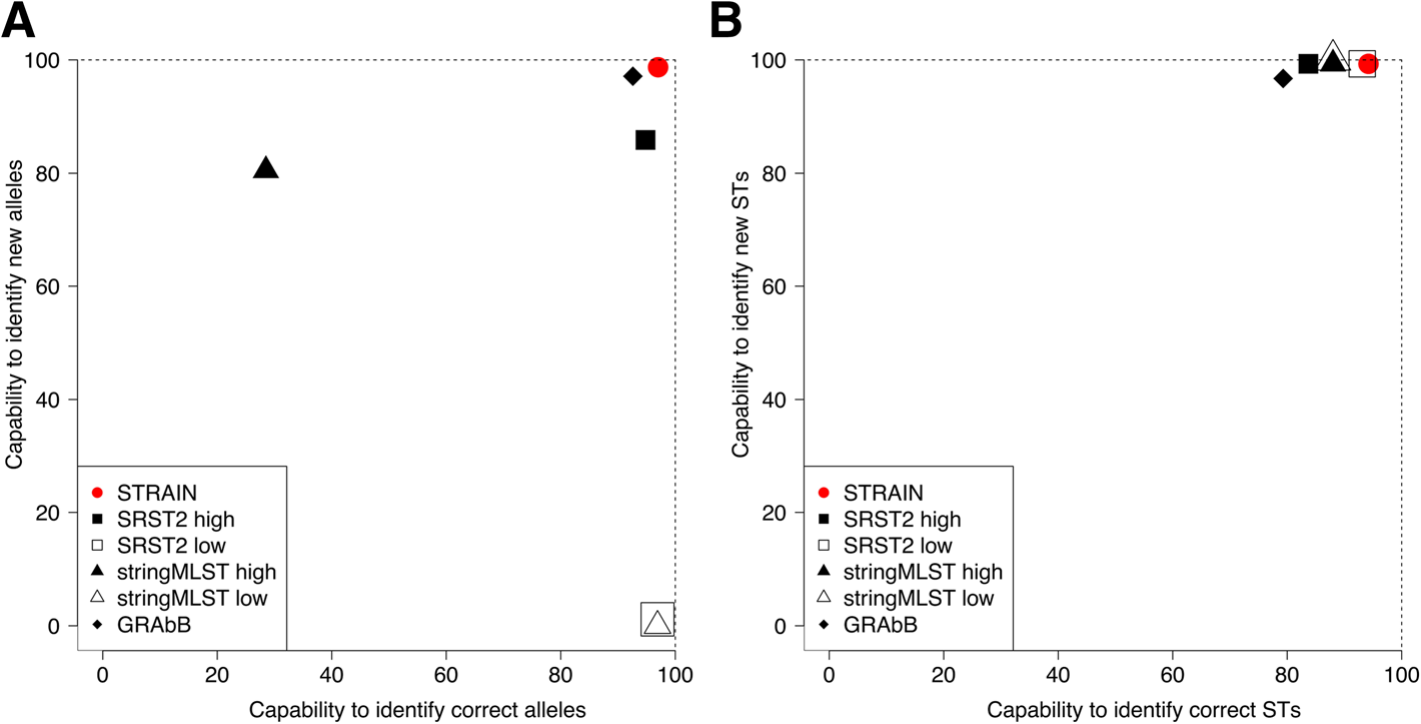
Comparison of STRAIN, SRST2, stringMLST and GRAbB performances. A) Capability to identify correct alleles vs capability to identify new alleles B) Capability to identify correct STs vs capability to to identify new STs

Accounting together the values of capability to identify correct and new alleles achieved by each tool –where possible considering different stringency filters (high and low)- STRAIN shows the best combination, as summarized graphically in Fig. 2 where x-axis represents capability to identify correct alleles and y-axis capability to identify new alleles.

### Preprocessing of reads does not affect STRAIN capability to identify correct alleles

To test if preprocessing of reads – trimming – could further improve the sensitivity of the programs at allele level, we run them on the benchmark dataset preprocessed by Trimmomatic [9].

As shown in Table 4 the preprocessing doesn’t seem to be a step necessary to improve the performance of STRAIN. Instead we noticed an effect, both positive and negative, introduced by the trimming of reads for the other tested programs. GRAbB has not been tested since in its current release doesn’t allow in input reads of different lengths.

**Table 4:** performances measured on trimmed datasets

Software	Capability to identify correct alleles without trimming	Capability to identify correct alleles with trimming	Difference
STRAIN	**97.0% (3666/3780)**	**97.0% (3666/3780)**	**=**
SRST2 high stringency	94.8% (3583/3780)	96.0% (3630/3780)	+
SRST2 low stringency	96.9% (3662/3780)	96.6% (3655/3780)	–
stringMLST high stringency	28.5% (1076/3780)	24.1% (911/3738)	–
stringMLST low stringency	96.9% (3664/3780)	96.2% (3638/3738)	–

Capability to identify correct alleles without and with trimming pre-processing of the reads. In Difference column “=” means no difference, + and - mean increased and decreased capability, respectively. In bold the maximum value

### By setting up parallel computation STRAIN drastically reduces its running time

STRAIN implementation leverages on embedded parallelism in R environment through package parallel. As shown in Additional file 3 Figure S1 Panel A, running time for STRAIN and the other programs increases with the total number of input reads. Further details on how running time has been calculated together with computational resource specifications are reported in Additional file 2, Section Running times. When STRAIN was run without parallelism, processing one allele at the time and with one thread for bowtie2, it showed a median of 3 h and 7 min (IQR = 51 min).

To measure the maximal reduction in running time provided by parallel computations, we reanalyzed the most computationally intense cases selecting for each one of the seven species present in our benchmark the sample with the maximum number of reads (median 10,136,800, IQR 8554499). Executed on a Linux machine equipped with 48 CPUs and 250 Gb of RAM (see further specification on Additional file 2), tuned to analyze 7 alleles in parallel and allowing 7 threads for Bowtie2, STRAIN dropped its running times to a median of 8 min and 13 s (decreasing running time by 23-fold) as shown in Additional file 3: Figure S1 Panel C.

As shown in Additional file 3: Figure S1 Panel A, median RAM consumption for STRAIN is low, 259 MB (IQR 39 Mb), and it is not influenced by readset sample size. Further details on how RAM peak for each program has been calculated are reported in Additional file 2, Section Peak of RAM.

### Other potential applications

STRAIN can be used beyond the analysis of MLST genes; provided by any other appropriate database of alleles, it can annotate any locus, i.e. drug resistance genes, ribosomal genes and antigens.

## Discussions and conclusions

STRAIN uses a hybrid strategy between read mapping and assembly to assign sequence types (STs) to bacterial isolates.

In terms of capability to identify correct alleles and capability to identify new alleles outperforms the existing typing methods. When compared to SRST2, stringMLST and GRAbB, it improves the proportion of correct allele assignments to the total number of isolates from a minimum of 28.5% to a maximum of 97%.

When tested simulating the presence of new alleles it was efficiently capable to detect the new variants. In comparison with the other programs, STRAIN improves the capability to identify new alleles from a minimum of 1.1% to a maximum of 98.7%.

Even if the existing tools (SRST2, stringMLST and GRAbB) show already high performances, STRAIN delivers yet a key improvement over them because it is able to increase concomitantly capability to identify correct alleles and capability to identify new alleles. This key improvement brings STRAIN towards the asymptote of 100% in both capabilities. Such an asymptote might be hard to be achieved because even golden standard PCR typing might be erroneous in a small fraction of cases. In fact, among the 540 samples constituting our benchmark, 450 had a whole-genome assembly of the reads deposited in publicly available databases (PubMLST at http://pubmlst.org or Enterobase at https://enterobase.warwick.ac.uk) and the ST assignment by BLASTN against reference alleles databases was possible. Out of the 450 isolates, 10 (2.2%) resulted to have exactly the same ST assignment with all the tested typing tools based on reads but different to the one assigned by the PCR or capillary sequencing, pointing out the problem to ascertain who is actually measuring the real sample ST.

A distinct step in STRAIN analysis is represented by its *locus-wise* assembly strategy. For this reason, we decided to evaluate the software GRAbB, designed to selectively assembly genomic regions. Respect to GRAbB, STRAIN improves the capability to identify correct alleles from 92.6 to 97% and the capability to identify new alleles from 97.1 to 98.7%.

The sequences of the new detected alleles are reported by STRAIN together with the output for further evaluations or eventual submission to dedicated reference repositories. Being database driven, STRAIN, might be used to type also other loci beyond MLST, provided appropriate databases are used as input.

STRAIN depends on very few external programs, commonly present in the toolbox of end-users working on short read sequencing data. Its implementation as an R package together with a containerized solution in Docker makes it very easy to install and use.

## Additional files

Additional file 1: **Table.** containing for each of the 540 isolates used in the present study the SRA sample accession number (i.e ERR016635), the species of origin, the expected ST, the total number of reads in the sample, the RAM peak (kB) and the running time (s) for each program. (XLSX 96 kb)

Additional file 2: File describing the running setup of the programs and the specifications of the computational resource used to run the programs. (PDF 3690 kb)

Additional file 3: Figure showing the peak RAM consumption (kB) and the running time (s) of the programs tested. Panel A) running time as function of total number of reads for the 540 samples when programs are run using 1 core with a 1 thread for bowtie2 for STRAIN and SRST2. On the right, boxplots of the running time for each program; Panel B) running time on 7 samples selected to have the maximum number of reads among the samples of the same species when STRAIN is run setting 7 cores and 7 threads for bowtie2 and SRST2 set with 48 threads for bowtie2. On the right, boxplots of the running times per program in the seven selected biggest samples; Panel C) peak RAM consumption as function of the total number of reads of the 540 samples and boxtplots for each program. (DOCX 21 kb)

## Data Availability

Project name: STRAIN. Project home page: https://gitlab.com/DalsaMat/STRAIN Docker: fpetrogalli:strain. Operating system(s): Linux/Mac. Requirements: Programs: SAMtools v1.2 or higher [10], bowtie2 [6] v2.2.6 or higher (open-source), bedtools2 2.25.0 [11], SPAdes [7] v3.6 or higher, Blast + [12] v2.3.0 or higher, R v3.2.3 or higher. R packages: seqinr v3.1–3, Biostrings v2.38.2, parallel v2.14.0, XML v3.98–1.3. Programming language: R. License: GPL. Any restrictions to use by non-academics**:** No.

## Abbreviations

MLST: Multi Locus Sequence Type
ST: Sequence Type
WGS: Whole Genome Sequencing
