# Supplementary figures and images for: STRAIN: an R package for multi-locus sequence typing from whole genome sequencing data
Source: BMC Bioinformatics. 2019 Nov 22;20(Suppl 9):347. doi: 10.1186/s12859-019-2887-1 (PMC6873635; doi:10.1186/s12859-019-2887-1)

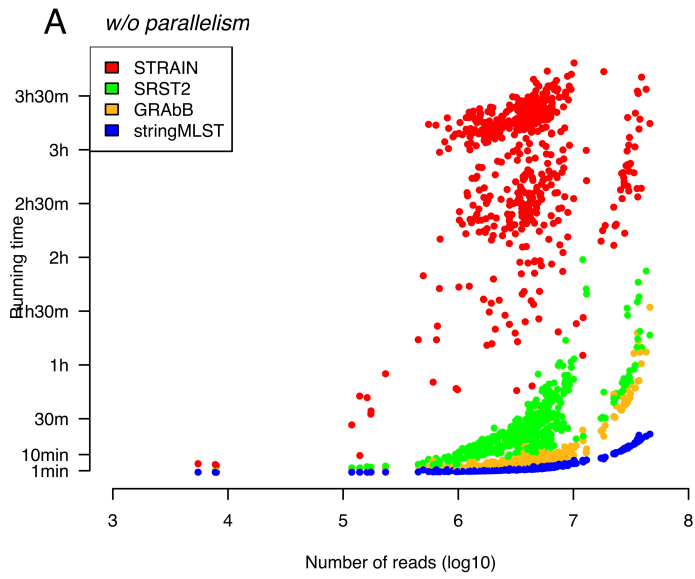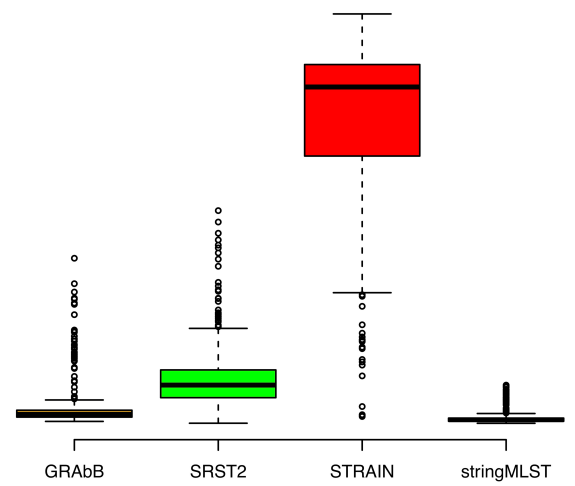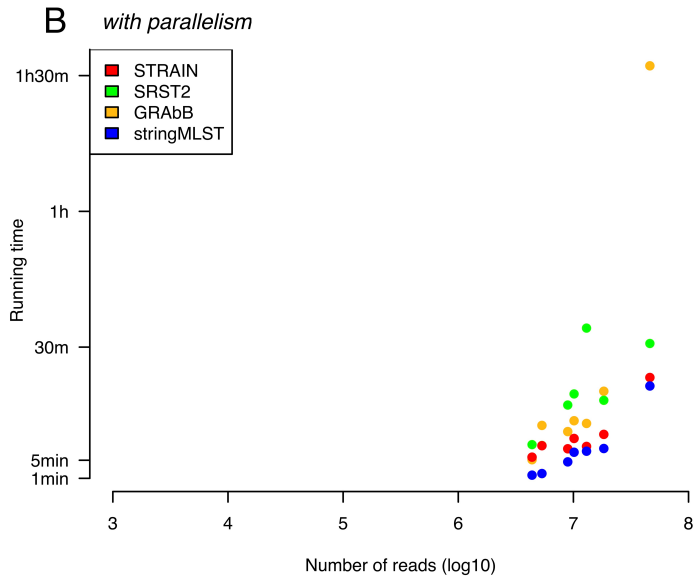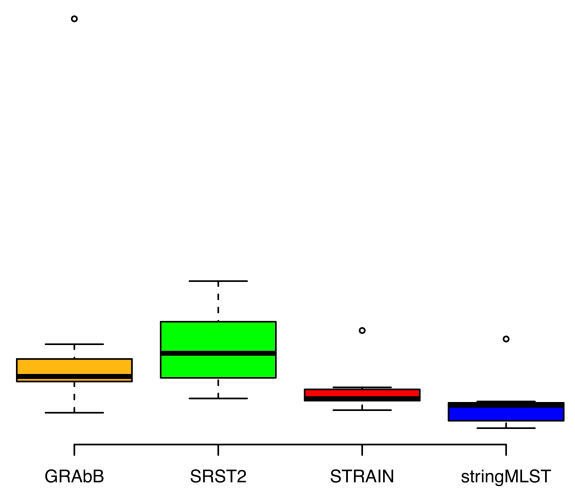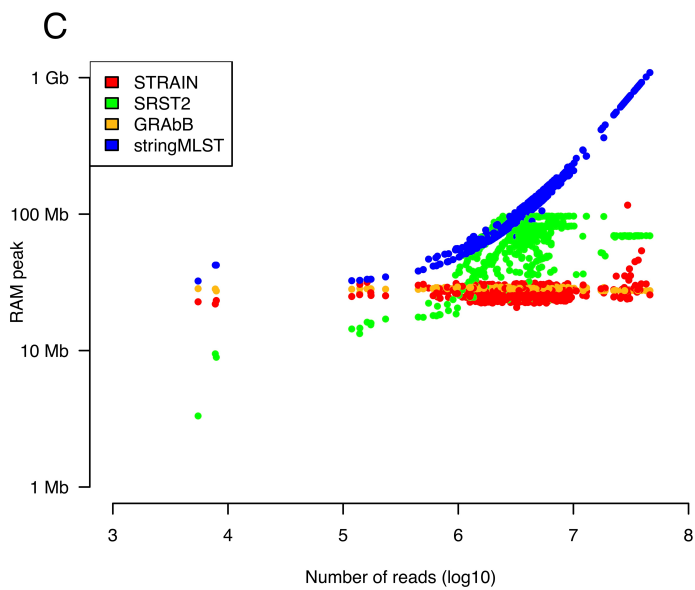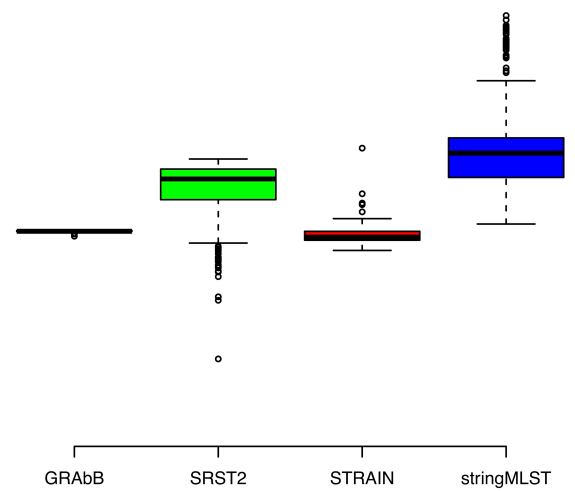

Supplement: Supplementary file 2 — File describing the running setup of the programs and the specifications of the computational resource used to run the programs. (PDF 3690 kb) [file 12859_2019_2887_MOESM2_ESM.pdf]
