# Supplementary material for: STRAIN: an R package for multi-locus sequence typing from whole genome sequencing data
Source: BMC Bioinformatics. 2019 Nov 22;20(Suppl 9):347. doi: 10.1186/s12859-019-2887-1 (PMC6873635; doi:10.1186/s12859-019-2887-1)
Supplement: Supplementary file 3 — Figure showing the peak RAM consumption (kB) and the running time (s) of the programs tested. Panel A) running time as function of total number of reads for the 540 samples when programs are run using 1 core with a 1 thread for bowtie2 for STRAIN and SRST2. On the right, boxplots of the running time for each program; Panel B) running time on 7 samples selected to have the maximum number of reads among the samples of the same species when STRAIN is run setting 7 cores and 7 threads for bowtie2 and SRST2 set with 48 threads for bowtie2. On the right, boxplots of the running times per program in the seven selected biggest samples; Panel C) peak RAM consumption as function of the total number of reads of the 540 samples and boxtplots for each program. (DOCX 21 kb) [file 12859_2019_2887_MOESM3_ESM.docx]

**STRAIN: an R package for multi-locus sequence typing from whole genome sequencing data.**

Mattia Dalsass^1,2^, Margherita Bodini^1^, Christophe Lambert^3^, Marie-Cécile Mortier^3^, Marco Romanelli^1,4^, Duccio Medini^1^, Alessandro Muzzi^1^, Alessandro Brozzi^1*^

^1^GSK, Siena, Italy; ^2^ Department of Experimental Oncology, European Institute of Oncology, Milan, Italy. ^3^ GSK, Rixensart, Belgium; ^4^ Università degli Studi di Siena, Italy

^*^To whom correspondence should be addressed

Supplementary Methods

SRST2 and stringMLST

SRST2 version 0.2 was run with default parameters.

stringMLST version 0.5.2 was run with k-mer of length 35 described as the best choice in the original article and default fuzzy parameter (300).

A divergence was noticed when comparing the sensitivity at ST level of SRST2 publication (94.4%) with the capability to identify correct STs obtained in our analysis (83.7% and 93.1% respectively with the high and low stringency). This might be due to several changes occurred since the SRST2 publication (2014):

- Reference allele databases have increased their sizes during the years;
- SRST2 has been updated from the original version to the 0.2 (used in our analysis);
- The read sets deposited at ENA have been updated before December 2017.

To ensure stringMLST was correctly installed, it has been tested on the original publication dataset (10 samples each from 4 species: *N*eisseria *meningitidis*, *Streptococcus pneumoniae*, *Campylobacter jejuni* and *Chlamydia trachomatis*). The results obtained in our local installation were identical to the ones reported in the manuscript.

GRAbB

We downloaded and installed GRAbB from the public repository at https://github.com/b-brankovics/grabb. The termination criterion used is the type “longest” requiring the longest contig of the assembly to reach a minimum length, in our case it was set as the allele length with additional 50bp upstream and downstream.

Trimmomatic

Read quality trimming was performed through Trimmomatic v0.36. The parameters choosen are the following:

- 4-bases wide sliding window;
- Minimum average quality per base = 20;
- Leading low quality = 3;
- Trailing low quality = 3;
- Minimum length: 25 bp.

*Supplementary Tables*

Supplementary Table S1. **Comparison of capability to identify correct ST of STRAIN, SRST2, stringMLST and GRAbB.** Summary of percentage of no output and capability to identify correct STs.

| **Software** | **Percentage of no output** | **Capability to identify correct ST** |
| --- | --- | --- |
| STRAIN (ST) | 0.07%  (4/540) | **94.2%**  **(509/540)** |
| SRST2  high stringency (ST) | 0.07%  (4/540) | 83.7%  (452/540) |
| SRST2  low stringency (ST) | 0.07%  (4/540) | 93.1%  (503/540) |
| stringMLST  (high and low stringency, ST) | 0%  0/540 | 88%  (475/540) |
| GRAbB (ST) | 0.3%  (18/540) | 79.3%  (428/540) |

Supplementary Table S2. **Comparison of capability to identify new STs of STRAIN, SRST2, stringMLST and GRAbB.** Summary of percentage of no output and capability to identify new STs.

| **Software** | **Percentage of no output** | **Capability to identify new STs** |
| --- | --- | --- |
| STRAIN (ST) | 0.07%  (4/540) | 99.3%  (536/540) |
| SRST2  high stringency (ST) | 0.07%  (4/540) | 99.3%  (536/540) |
| SRST2  low stringency (ST) | 0.07%  (4/540) | 99.3%  (536/540) |
| stringMLST  (high and low stringency, ST) | 0%  0/540 | **100%**  **540/540** |
| GRAbB (ST) | 0.3%  (18/540) | 96.7%  (522/540) |

*Specification of the computational resource*

Ubuntu 12.04.5 LTS (GNU/Linux 3.5.0-54-generic x86_64).

48 cores, 4x AMD Opteron(tm) Processor 6168 @ 1.90 GHz

250 Gb RAM

*Running time*

Running time was measured by /usr/bin/time -v command extracting the field Elapsed (wall clock) time.

Run on a single core, allowing a single thread for bowtie2 in SRST2 and STRAIN, stringMLST resulted to be the fastest with a median running time of 126 seconds (IQR 103 s). As shown in Supplementary Figure 1 Panel A, a clear trend is seen with running time increasing as long as the number of reads present in the sample. Even if stringMLST is not implemented in its current release to be parallelized, its running times are already very low. On the contrary SRST2 might be launched allowing multiple threads for bowtie2. In Panel B, SRST2 on the seven samples with highest number reads per species has been run with 48 threads and reached a median of 18 minutes (IQR = 12), STRAIN reached a median of 8 minutes (IQR = 2.4).

*Peak of RAM*

Peak RAM consumption was calculated adapting memusg bash script (https://gist.github.com/netj/526585) to allow the tracking of all child processes.

In general peaks of RAM consumption are low for all the programs and don’t exceed 10 Gb (stringMLST). We noticed a strong increasing trend for stringMLST with the number of reads. Also SRST2 shows an increasing trend while STRAIN and GRAbB are almost flat. Although all the programs are compatible with modern servers, the last two can be run easily even on a personal computer, making multi-locus sequence typing more accessible to basic users.
